# Novel Bacterial Production of Two Different Bioactive Forms of Human Stem-Cell Factor

**DOI:** 10.3390/ijms22126361

**Published:** 2021-06-14

**Authors:** Eunyoung Lee, Michelle Novais de Paula, Sangki Baek, Huynh Kim Khanh Ta, Minh Tan Nguyen, Taeck-Hyun Jeong, Chong Jai Kim, Yeon Jin Jang, Han Choe

**Affiliations:** 1 Department of Physiology, Bio-Medical Institute of Technology, University of Ulsan College of Medicine, Asan Medical Center, Seoul 05505, Korea; dmsudjk79@gmail.com (E.L.); michellenovais.ko@gmail.com (M.N.d.P.); sangkib64@gmail.com (S.B.); khanhta3103@gmail.com (H.K.K.T.); minhtannguyen279@gmail.com (M.T.N.); taeckja@naver.com (T.-H.J.); yjjang@amc.seoul.kr (Y.J.J.); 2 Department of Pathology, Asan-Minnesota Institute for Innovating Transplantation, University of Ulsan College of Medicine, Asan Medical Center, Seoul 05505, Korea; ckim@amc.seoul.kr

**Keywords:** human stem-cell factor, hSCF248, hSCF164, MBP, recombinant protein, soluble protein

## Abstract

Human stem-cell factor (hSCF) stimulates the survival, proliferation, and differentiation of hematopoietic cells by binding to the c-Kit receptor. Various applications of hSCF require the efficient and reliable production of hSCF. hSCF exists in three forms: as two membrane-spanning proteins hSCF248 and hSCF229 and truncated soluble *N*-terminal protein hSCF164. hSCF164 is known to be insoluble when expressed in *Escherichia coli* cytoplasm, requiring a complex refolding procedure. The activity of hSCF248 has never been studied. Here, we investigated novel production methods for recombinant hSCF164 and hSCF248 without the refolding process. To increase the solubility of hSCF164, maltose-binding protein (MBP) and protein disulfide isomerase b’a’ domain (PDIb’a’) tags were attached to the *N*-terminus of hSCF164. These fusion proteins were overexpressed in soluble form in the Origami 2(DE3) *E. coli* strain. These solubilization effects were enhanced at a low temperature. His-hSCF248, the poly-His tagged form of hSCF248, was expressed in a highly soluble form without a solubilization tag protein, which was unexpected because His-hSCF248 contains a transmembrane domain. hSCF164 was purified using affinity and ion-exchange chromatography, and His-hSCF248 was purified by ion-exchange and gel filtration chromatography. The purified proteins stimulated the proliferation of TF-1 cells. Interestingly, the EC_50_ value of His-hSCF248 was 1 pg/mL, 100-fold lower than 9 ng/mL hSCF164. Additionally, His-hSCF248 decreased the doubling time, increased the proportion of S and G2/M stages in the cell cycle, and increased the c-Myc expression at a 1000-fold lower concentration than hSCF164. In conclusion, His-hSCF248 was expressed in a soluble form in *E. coli* and had stronger activity than hSCF164. The molecular chaperone, MBP, enabled the soluble overexpression of hSCF164.

## 1. Introduction

Human stem-cell factor (hSCF), also known as the c-Kit ligand, mast cell growth factor, or steel factor, stimulates hematopoiesis, stem-cell maintenance, gametogenesis, mast-cell development, and melanogenesis [1,2,3,4,5,6]. hSCF has been used for the differentiation of stem cells into a range of specialized cells, such as endothelial cells, erythropoietic cells, lymphoid progenitors, osteoclasts, and spermatozoa [7]. Primordial germ cells have been reported to be induced from human pluripotent stem cells by using hSCF. hSCF has also been used in the generation of humanized mice [8]. Furthermore, hSCF has assisted in the recovery of cardiac function following myocardial infarction by increasing the number of cardiomyocytes and vascular channels [9]. Therefore, the efficient production of bioactive hSCF is desirable for multiple applications.

**Figure 1:**
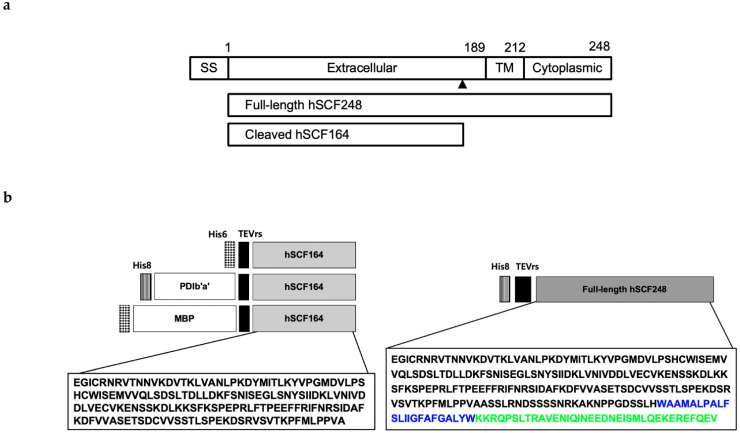
Design of hSCF164 and hSCF248 constructs. (**a**) Structure of His-hSCF248 and hSCF164. The arrow indicates the proteolytic cleavage site. SS, signal sequence; TM, transmembrane. (**b**) Schematic structure of the His-hSCF248, His-hSCF164, PDIb’a’-hSCF164, and MBP-hSCF164 constructs. The amino-acid sequence in black is the extracellular region, the blue sequence is the membrane penetration region, and the green sequence is the cytoplasmic region. MBP, maltose-binding protein; PDIb’a’, b’a’ domain of protein disulfide isomerase; TEVrs, tobacco etch virus protease restriction site; His6, six histidines; His8, eight histidines.

Splicing of hSCF mRNA produces two isoforms (His-hSCF248 and hSCF220) [10,11,12]. Both isoforms are membrane proteins with a single transmembrane (TM) domain. The longer His-hSCF248 contains a proteolytic cleavage site such that the truncated *N*-terminal hSCF (hSCF164) can be released from the extracellular domain of the protein (Figure 1a) [12,13,14,15]. At a low concentration similar to the physiological condition, hSCF164 exists mostly as a monomer [16]. The *N*-terminal 141 residues of hSCF have been identified as a functional core, which includes the dimer interface and portions that bind and activate the c-Kit receptor [17]. hSCF164 forms a noncovalently associated homodimer when it interacts with c-Kit to trigger receptor dimerization and signaling [18,19]. The hSCF contains four *N*-linked and three *O*-linked glycosylation sites [15,19]. However, glycosylation is not involved in the binding to the receptor [20,21].

Many studies of hSCF production have focused on hSCF164. In early studies, eukaryotic expression systems were used in an attempt to produce hSCF164 [22,23,24]. However, difficulties remain in the use of this system and in the comparatively high cost. For the more economic and efficient production of hSCF164, heterologous expression using *Escherichia* *coli* has been adopted [13,25,26,27,28,29,30]. However, hSCF164 expressed in *E. coli* resulted in denatured proteins and inclusion bodies. This may be caused by two disulfide bonds that are essential for maintaining the hSCF164 structure [25]; however, the cytoplasm of *E. coli* is a reducing environment that prevents the formation of disulfide bonds. These denatured proteins require a cumbersome solubilization and refolding process, and, even after refolding, the biological activity can be attenuated. Thioredoxin has been used as a fusion partner or co-expressed for the soluble expression of hSCF164 in *E. coli* cytoplasm [31,32].

This study aimed to optimize the high solubility and expression conditions of bioactive hSCF164 and His-hSCF248 in *E. coli* without refolding, thereby enabling more economic and efficient production. To achieve this, we connected solubilization tags to the hSCF164 and induced protein expression at low temperatures in genetically engineered *E. coli* strains. We also expressed His-hSCF248 in different *E. coli* strains. Both proteins were purified using several chromatography techniques. Lastly, we assessed the biological activities of the purified proteins using human erythroleukemia TF-1 cell line. To the best of our knowledge, this is the first time the activity of His-hSCF248 has been studied.

## 2. Results

### 2.1. Design of Expression Plasmids

Three constructs and one construct for expression in *E. coli* were designed for hSCF164 and hSCF248, respectively (Figure 1b). The signal sequence was excluded for cytoplasmic expression. Two solubility enhancer tag proteins, MBP and PDIb’a’, were used for hSCF164. The His tag was placed at the front of all the constructs for easy purification. The tobacco etch virus protease restriction site (TEVrs), ENLYFQG, was placed between the tag and hSCFs to remove the tag during the purification. The constructs were named His-hSCF164, PDIb’a’-hSCF164, MBP-hSCF164, and His-hSCF248 (Figure 1b). The genes were codon-optimized for *E. coli* expression. Multisite Gateway cloning was used for easy cloning. Therefore, there were 12 additional amino acids, TTLYTKVVGGSG, a byproduct of the Gateway cloning, between the tag proteins and the TEVrs.

### 2.2. Expression and Solubility of the Recombinant Proteins

**Figure 2:**
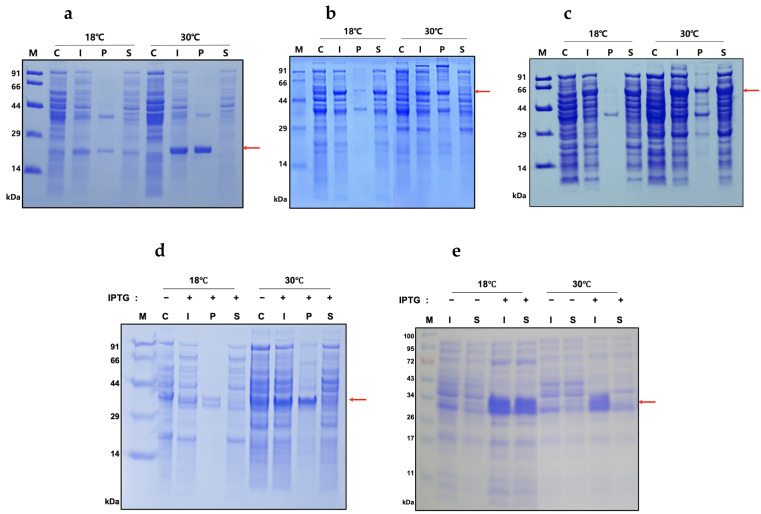
Expression and solubility analysis of fusion protein in *E. coli*. (**a**) His-hSCF164, (**b**) PDIb’a’-hSCF164, and (**c**) MBP-hSCF164 plasmids were transformed into Origami 2(DE3) strain, and the fusion proteins were expressed. His-hSCF248 plasmid was transformed into (**d**) Origami 2(DE3) and (**e**) BL21(DE3) strain, and the protein was expressed. Protein expression was induced by 0.5 mM IPTG at 18 °C and 30 °C. The red arrows indicate the His-hSCF164 (22 kDa), PDIb’a’-hSCF164 (52.6 kDa), MBP-hSCF164 (62.4 kDa), and His-hSCF248 (31.9 kDa). M, molecular weight size marker; C, total proteins before induction; I, total protein after induction; P, insoluble portion after cell lysis; S, soluble supernatant after cell lysis.

The plasmids were transformed into the *E. coli* strain Origami 2(DE3), and the expressions of the proteins were induced at two different temperatures of 18 °C and 30 °C. After the disruption of cells by sonication, the expression levels and the solubilities were analyzed using SDS-PAGE (Figure 2, Table 1 and Table 2). The expression levels of His-hSCF164 were approximately 16% and 38% at 18 °C and 30 °C, respectively. The MBP and PDIb’a’ tags increased the expression level at 18 °C by more than twofold. The solubilities of His-hSCF164 were approximately 39% and 12% at 18 °C and 30 °C, respectively. MBP and PDIb’a’ tags increased the solubilities at 18 °C by more than twofold again. Interestingly, MBP tag also dramatically increased the solubilities at 30 °C. Therefore, considering the high expression and solubility levels, we chose to induce MBP-hSCF164 at 18 °C for further purification and functional analysis. 

The expression level and the solubility level of His-hSCF248 in Origami 2(DE3) were relatively low (Figure 2d). Therefore, the BL21(DE3) *E. coli* strain was tested (Figure 2e). The expression level of His-hSCF248 in BL21(DE3) was increased at both temperatures, and the solubility was increased at 18 °C but not at 30 °C (Table 2). Therefore, BL21(DE3) was used for the purification of His-hSCF248.

### 2.3. Purification of hSCF164

**Figure 3:**
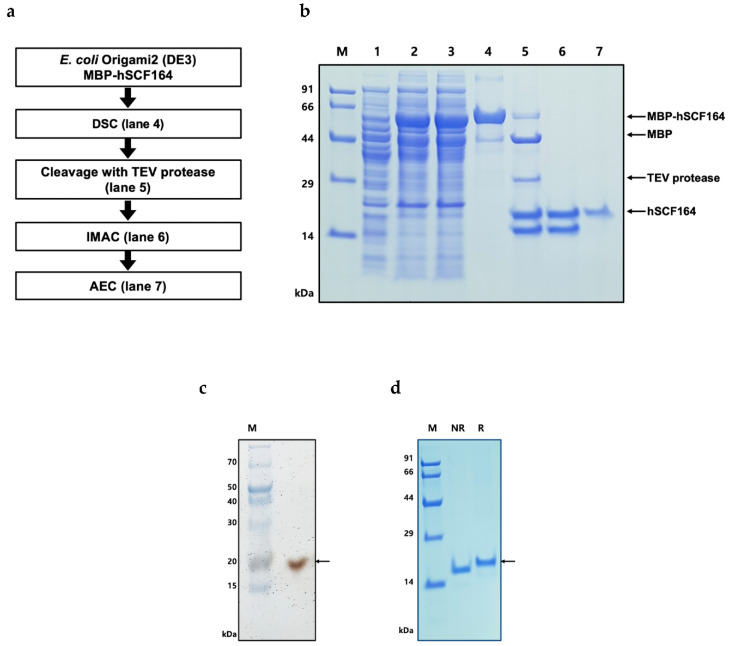
Purification of hSCF164 from MBP-hSCF164 in *E. coli*. (**a**) Flow chart of the purification steps. (**b**) hSCF164 was purified from MBP-hSCF expressed in *E. coli* Origami 2(DE3) using Dextrin Sepharose chromatography (DSC), immobilized metal affinity chromatography (IMAC), and anion-exchange chromatography (AEC). M, molecular weight size marker (homemade marker); lane 1, total cell proteins before IPTG induction as negative control; lane 2, total proteins induced by IPTG after cell sonication; lane 3, soluble proteins after cell sonication from total cell proteins; lane 4, MBP-hSCF fusion protein (62.4 kDa) purified with affinity chromatography; lane 5, result of TEV protease cleavage (28.6 kDa): MBP tag (43.9 kDa) and hSCF (18 kDa); lane 6, IMAC purification of hSCF164 after TEV cleavage; lane 7, hSCF164 (18 kDa) purified with ion-exchange chromatography. (**c**) Silver-stained gel under reducing conditions. M, molecular weight size marker. (**d**) Final purified hSCF164 under reducing and nonreducing conditions. M, molecular weight size marker (homemade marker); R, reducing; NR, nonreducing.

To acquire hSCF164 from MBP-hSCF164, a three-step purification process was performed (Figure 3a). *E. coli* cells expressing MBP-hSCF164 were sonicated, and the supernatant containing the fusion protein was obtained through centrifugation (Figure 3b, Lane 2,3). The fusion proteins were bound to a Dextrin Sepharose column (DSC) and were eluted with some impurities (Figure 3b, Lane 4). The purity was as low as 86% (Table 3). Digestion with TEV protease was performed in the buffer without buffer exchange to minimize the steps in the purification process. Several ratios (20:1, 10:1, and 5:1) for MBP-hSCF164:TEV protease at 18 °C for 18 h were used, with the 5:1 ratio producing the best result (data not shown). Approximately 85% of the fusion protein was cleaved by TEV protease (Figure 3b, Lane 5). In the SDS-PAGE gel, a truncated version of the hSCF164 band appeared after TEV cleavage. To remove the MBP-hSCF164, the tag protein, and TEV protease, an immobilized metal affinity chromatography (IMAC) column was utilized (Figure 3b, Lane 6). However, the smaller protein could not be removed by the IMAC column and, hence, required further purification. Considering the property of pI = 5.08 of the target protein, an anion Q column was used. The target protein was eluted with a smaller fragment at a similar concentration of NaCl. These proteins were separated by slowly raising the concentration of NaCl from 0 to 30% over 10 column volumes (CVs). In addition, unseparated fractions were purified again using a Q column so that more protein could be obtained. SDS-PAGE analysis showed a highly purified 18 kDa band of hSCF164 (Figure 3b, lane 7). The purity of the final protein was estimated by silver staining to be 99% under reducing conditions (Figure 3c). Nonreduced hSCF164 moved more quickly during electrophoresis, and no dimer size protein was detected (Figure 3d). Triton X-114 was added to the final product to remove endotoxins. The free endotoxin level was 0.15 EU/μg. Finally, 0.2 mg of pure hSCF164 was obtained from 500 mL of flask culture with a yield of 12.6% (Table 3).

### 2.4. Purification of Full-Length His-hSCF248

**Figure 4:**
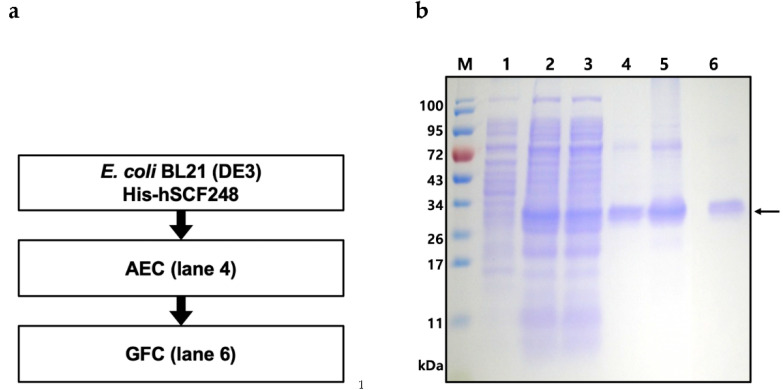
Purification of full-length His-hSCF248 from *E. coli*. (**a**) Flow chart of the purification steps. AEC, anion-exchange chromatography; GFC, gel filtration chromatography. (**b**) His-hSCF248 was purified using AEC and GFC. M, molecular weight size marker; lane 1, total cell proteins before IPTG induction as negative; lane 2, total proteins induced by IPTG after cell sonication; lane 3, soluble proteins after cell sonication from total cell proteins; lane 4, His-hSCF248 (31.9 kDa) purified with AEC; lane 5, concentrated His-hSCF248; lane 6, His-hSCF248 purified with GFC.

Soluble overexpressed His-hSCF248 was successfully purified using a relatively high pH buffer condition and two types of chromatography (Figure 4a). Because the construct contains a His tag, we initially tried to purify the protein using IMAC. However, the protein did not bind to the column (data not shown), even though we confirmed the presence of the His tag by both mass spectrometry analysis and Western blot (data not shown). Therefore, we turned to ion-exchange chromatography. Prior to the application of the ion-exchange column, we estimated the stability of the His-hSCF248 at different pH levels (from pH 3 to pH 10). His-hSCF248 was stable between pH 5 and 10 and precipitated at pH 3 and 4 (data not shown). After applying clarified lysate to a strong anion-exchange chromatography column, His-hSCF248 was eluted along with some impurities at pH 10 (Figure 4b, Lanes 4 and 5). The fusion protein was treated with TEV protease to remove the His8 tag. However, the His-tag was not cleaved (data not shown). After the next stage of purification using a gel permeation chromatography (GPC) column, purified protein was obtained (Figure 4b, Lane 6). The final protein purity was quantified as 96%. As a result, 2.4 mg of pure His-hSCF248 was obtained from 500 mL of flask culture with a yield of 23.9% (Table 4).

### 2.5. Effect of hSCF164 and His-hSCF248 on Cellular Proliferation 

**Figure 5:**
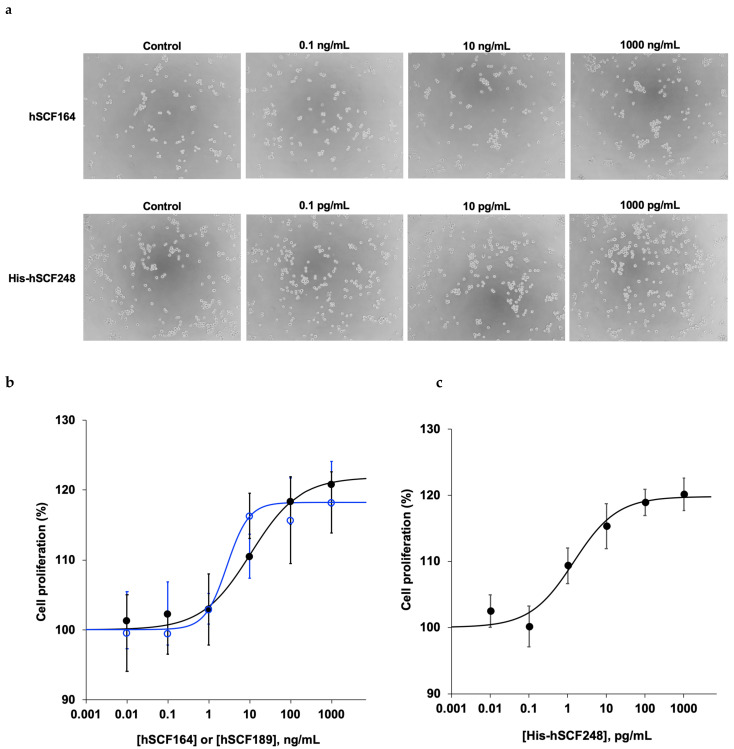
TF-1 cell proliferation assays of commercial hSCF164, and purified hSCF164 and His-hSCF248. (**a**) Microscope images of TF-1 cell growth that was promoted by treatment with purified hSCF164 and His-hSCF248 for 12 h. (**b**) Dose-dependent cell proliferation by hSCF164 (black closed circle) and commercial hSCF189 (blue open circle). (**c**) Dose-dependent cell proliferation by His-hSCF248.

The biological activities of purified hSCF164 and His-hSCF248 were evaluated on the basis of their ability to stimulate proliferation of TF-1 cells in vitro. To understand the influence of fetal bovine serum (FBS), the preliminary activity test of purified protein was performed under different FBS concentrations (0%, 1%, and 10%); 10% FBS produced the best condition for the activity, whereas there was no cell proliferation in serum-free conditions (data not shown). Both hSCF164 and His-hSCF248 proteins activated the growth of TF-1 cells in a dose-dependent manner (Figure 5a). TF-1 cell proliferation was significantly increased after treatment with 100 and 1000 ng/mL hSCF164 compared with that in the control (*p* < 0.05) (Figure 5b). The EC_50_ value of hSCF164 was 10.6 ng/mL (6.5 nM); the Hill coefficient for the stimulation was 0.73. For the commercial hSCF189, i.e., the whole extracellular domain produced from HEK293 cells, the EC_50_ and the Hill coefficient were 2.8 ng/mL (0.13 nM) and 1.6, respectively. TF-1 cell proliferation was also significantly increased after treatment with 1000 pg/mL His-hSCF248 compared with control (*p* < 0.05) (Figure 5c). Interestingly, the EC_50_ value of His-hSCF248 was 1 pg/mL (0.32 pM), with a Hill coefficient of 1.21. These results indicated that hSCF164 and hSCSF248 proteins produced in this study stimulated cell proliferation and that our purified His-hSCF248 was almost 400-fold more efficient than commercial hSCF189 in terms of molar concentration. Additionally, the noncleaved MBP-hSCF164 showed slight activity, and the mixture of the two cleaved proteins (Figure 3b, lane 6) showed no activity (data not shown).

**Figure 6:**
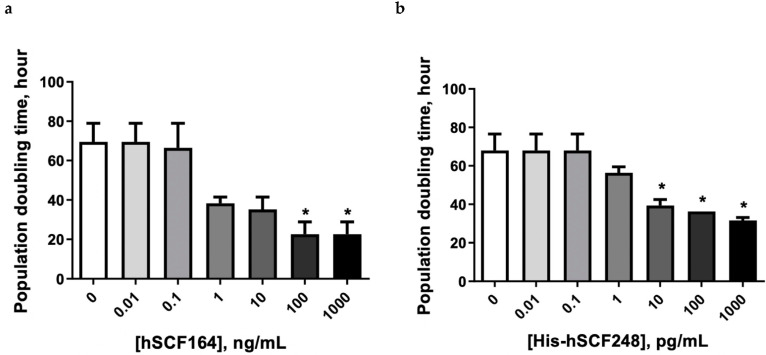
hSCF164 and His-hSCF248 effect on doubling time of TF-1 cells. Dose-dependent cell doubling time (h) was changed by treatment with hSCF164 (**a**) and His-hSCF248 (**b**). Data are presented as the mean ± SEM (*n* = 4). One-way ANOVA and Dunnett post hoc test (* *p* < 0.05).

As shown in Figure 6, the population doubling time of TF-1 cells treated with hSCF164 and His-hSCF248 decreased compared with that in untreated cells. The doubling time in the 100 ng/mL hSCF164-treated group significantly decreased compared with that of the control (*p* < 0.01), and the doubling times of the 10, 100, and 1000 pg/mL His-hSCF248-treated groups significantly decreased compared with that of the control (*p* < 0.01). 

### 2.6. Effect of hSCF164 and His-hSCF248 on Cell Cycle

**Figure 7:**
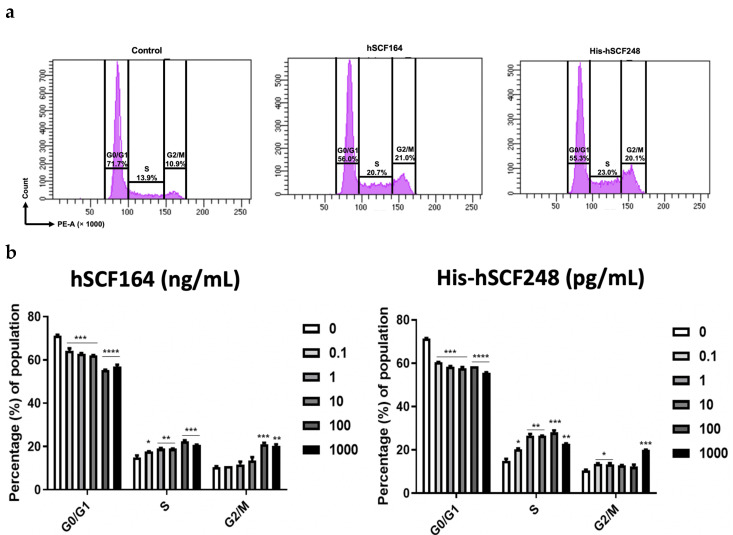
Flow cytometric analysis of cell cycle following treatment with hSCF. (**a**) Cell cycles of treated TF-1 cells were analyzed with FACS. The concentrations of hSCF164 and His-hSCF248 were 1 μg/mL and 1 ng/mL, respectively. (**b**) The relative percentages of cell cycle phase by hSCF164 and His-hSCF248 were dose-dependent. Note that the units of concentration for hSCF164 and His-hSCF248 were ng/mL and pg/mL, respectively. One-way ANOVA and Dunnett post hoc (* *p* < 0.05, ** *p* < 0.01, *** *p* < 0.001, **** *p* < 0.0001).

The cell cycle of TF-1 cells in the presence of hSCF164 and His-hSCF248 was analyzed using FACS (Figure 7a). The frequency of TF-1 cells treated with hSCF164 was significantly decreased in the G0/G1 stage and increased in the S stage (Figure 7b). The frequency of cells in the G2/M phase was significantly increased with 1000 ng/mL hSCF164 treatment. As shown in Figure 7a, when compared with that with the control, the ratio of cells in the G0/G1 stage treated with 1000 ng/mL hSCF164 g decreased from 71.7 to 56.0% but increased in the S and G2/M stages from 13.9 to 20.7% and 10.9 to 21%, respectively. In the His-hSCF248 treatment group, the ratio of cells was significantly decreased in the G0/G1 stage but was increased in the S and G2/M stages. As shown in Figure 7a, when compared with that of the control, the ratio of cells in the G0/G1 stage with 1000 pg/mL His-hSCF248 treatment decreased from 71.7 to 55.3% but increased in the S and G2/M stages from 13 to 23% and 10.9 to 20.1%, respectively.

### 2.7. hSCF Regulates Gene Expression Involved in Cell Growth

**Figure 8:**
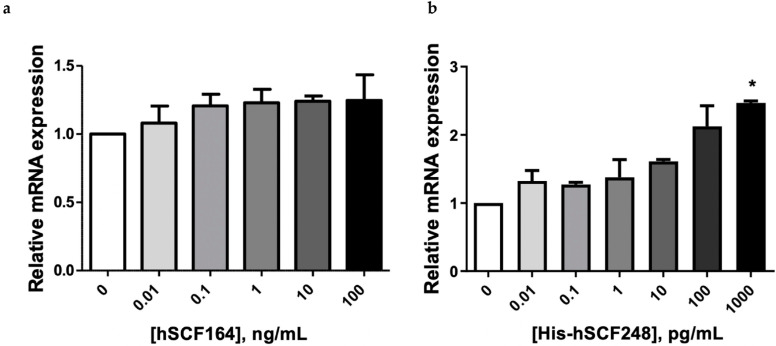
Expression levels of *c-Myc* mRNA by treatment with hSCF164 and His-hSCF248 in TF-1 cell line. Dose-dependent gene expression changes by treatment with hSCF164 (**a**) and His-hSCF248 (**b**). Gene expression was normalized to RPL mRNA expression. Data are presented as the mean ± SEM (*n* = 4). One-way ANOVA and Dunnett post hoc (* *p* < 0.05).

The expression change of the gene upon hSCF treatment was examined by real-time PCR. The expression of *c-Myc* mRNA was increased in cells treated with hSCF164 for 12 h (Figure 8a). Treatment of TF-1 cells with His-hSCF248 more significantly increased expression of *c-Myc* mRNA (Figure 8b). Note that the units of concentration differ by a factor of 1000. 

## 3. Discussion

A surprising finding in this study is that hSCF248 with its transmembrane domain can be expressed in a highly soluble form in *E. coli* cytoplasm, and a more surprising finding is that hSCF248 has much stronger biological activity than hSCF164. Previously, most structural and functional studies of hSCF focused on hSCF164, i.e., the released form. Additionally, only hSCF164 or hSCF189 protein is commercially available. As far as we are aware, there has been no report of prokaryotic expression or cytokine-like functions of hSCF248. In the present study, the His-hSCF248 used contains a hydrophobic transmembrane domain that interferes with proper folding without membrane. It is unknown how His-hSCF248 is so soluble. Usually, the effect of EC_50_ of hSCF164 on TF-1 cell proliferation is reported to be ~10 ng/mL [31,32,33]. Consistent with these results, our purified hSCF164 and commercial hSCF189 showed EC_50_ values of 2–10 ng/mL. The commercial hSCF189 was produced from a mammalian cell line so that the disulfide bonds and glycosylation were more likely to be present and the biological activities were similar to each other, validating our assay system, as well as our purified hSCF164 protein. The effect of His-hSCF248 on the TF-1 cell proliferation is 400-fold stronger than that of commercial hSCF189 in terms of molar concentration. The hSCF164 induces gene expression regulation related to cell growth by activating the c-Kit receptor and downstream signaling pathway [34,35,36]. His-hSCF248 demonstrated strong activity at 1000-fold lower concentration than hSCF164 in c-Myc mRNA expression, doubling time, and cell cycle. The stronger biological activity of His-hSCF248 is difficult to explain. One possibility is that His-hSCF248 is more easily dimerize than hSCF164. hSCF is a monomer at low concentration similar to physiological conditions [16]. When it binds to and activates its receptor, it is a dimer form [18,19].

*E. coli* is one of the most widely used bacterial hosts for high-level recombinant protein production [37]. The cytoplasm of *E. coli* is generally a reducing environment; therefore, many recombinant proteins with disulfide bonds are produced in insoluble forms [38]. hSCF164 has also been expressed in misfolded insoluble form in *E. coli*, resulting in inclusion bodies requiring complicated refolding processes to produce an active form [26,28,39]. MBP and PDIb’a’ are two tags that have proven to be consistent effective solubilization enhancers [40,41]. Therefore, the two tags were tested for their effects on the solubility of the hSCF164. Indeed, the two tags increased the solubility of hSCF164 significantly compared to the His-tagged hSCF164 (Figure 2 and Table 1 and Table 2). The mechanism underlying these positive solubilization effects of the MBP and PDIb’a’ tags is not completely understood. 

Solubility of proteins was increased when expression was induced at low temperatures [42,43,44,45]. In our study, hSCF164 and His-hSCF248 were each induced at 18 °C and at 30 °C to investigate the temperature effect on the solubility of the proteins. Both proteins showed higher solubility when induced at the lower temperature (Figure 2). This suggests that the proteins were slowly produced at low temperatures, providing more time for correct folding [46,47,48]. 

During purification, the MBP tag from the MBP-hSCF164 was removed with TEV protease cleavage. The usual condition for the reaction of TEV protease is 50 mM Tris at room temperature [49]. After Dextrin Sepharose chromatography, the buffer contained 500 mM NaCl that would interfere with the TEV protease activity [49,50]. After testing several conditions, a favorable condition for rapid and simple digestion was identified. The sample mixture digested with the TEV protease underwent a degree of aggregation during dialysis for the next purification step. Protein precipitation and aggregation after tag removal led to a low protein yield, which is a frequent problem that remains to be solved [51,52]. This was addressed successfully here in two ways: one was to reduce the protein concentration by diluting the eluted sample with buffer A, and the other was to treat with TEV protease after the dialysis step. After the cleavage of the TEV protease site, a smaller band along with the hSCF164 protein was identified on the SDS-PAGE gel (Figure 3b, lane 6). Interestingly, this mixture did not have the stimulatory effect on TF-1 proliferation (data not shown). It is possible that the truncated hSCF164 interacted with hSCF164 to form a heterodimer, thereby inhibiting hSCF164. Only when hSCF164 was separated from the smaller protein did it show biological activity. It was difficult to isolate the smaller protein alone. The production yield of His-hSCF248 was 10-fold that of hSCF164 when they were purified from 500 mL of flask culture (Table 3 and Table 4).

TF-1 cells are an immortal cell line derived from human erythroleukemia (Kitamura, 1989, PMID: 2663885). According to the American Type Culture Collection, TF-1 cells respond to a variety of lymphokines and cytokines including hSCF. Most companies selling hSCF show the biological activities of their products using TF-1 cell proliferation. Furthermore, many researchers have used TF-1 cells to test the activity of hSCF [31,32,33]. Therefore, we chose TF-1 cells for our assay system.

## 4. Material and Methods

### 4.1. Materials

All chemicals were analytical grade. Reagents were acquired from the following suppliers: lambda integrase and excisionase from Elpis Biotech (Daejeon, Korea); dithiothreitol (DTT) and IPTG from Anaspec, Fremont, CA, USA); ampicillin and gentamicin from Duchefa Biochemie (Haarlem, The Netherlands); sodium chloride, glycerol, sodium cyanoborohydride, and sodium phosphate dibasic from Samchun Chemical (Pyeongtaek, Korea); Coomassie brilliant blue R-250 and Tris-HCl from Amresco (Solon, OH, USA); ammonium bicarbonate from Junsei Chemical (Tokyo, Japan); imidazole from Daejung Chemicals (Siheung, Korea); Triton X-114 from Sigma-Aldrich (St. Louis, MO, USA); GM-CSF from (Peprotech, Rocky Hill, NJ, USA). hSCF189 produced from HEK293 cells was obtained from ACROBiosystems (Newark, DE, USA). The Toxin Sensor Chromogenic LAL Endotoxin Assay Kit was from GenScript (Piscataway, NJ, USA), Cell Counting Kit-8 was from Dojindo (MD, USA), and RPMI-1640 medium, 0.25% trypsin-EDTA, FBS, and penicillin/streptomycin were from GIBCO (Carlsbad, CA, USA). The Silver Stain Plus kit was purchased from Bio-Rad Laboratories (Hercules, CA, USA). *E. coli* Origami 2(DE3) (DE3) cells were acquired from Novagen (Madison, WI, USA). Full-length His-hSCF248 gene was codon-optimized for *E. coli* expression and synthesized by Epoch life science (Missouri, TX, USA). The TF-1 cell line was obtained from ATCC (Manassas, VA, USA). All chromatography columns and an ÄKTA Prime and ÄKTA Start for purification were purchased from GE Healthcare (Piscataway, NJ, USA). The 0.45 µm pore size filter was from Hyundai Micron (Seoul, Korea). Dialysis membranes were from Viskase (Darien, IL, USA), and Amicon Ultra concentrators were from Merck Millipore (Billerica, MA, USA). Acrodisc syringe filters were acquired from Pall Korea (Seoul, Korea).

### 4.2. Construction of Plasmids

The hSCF-expressing vectors were constructed using Gateway cloning (Figure S1, Supplementary Materials). The gene for hSCF164 (residues 1 to 164) was amplified by PCR, using the codon-optimized and chemically synthesized gene as the template. The following primers were used for amplification: 5′–GGG GAC AAG TTT GTA CAA AAA AGC AGG CTT CGA AAA CCT GTA CTT CCA GGG CGA GGG TAT CTG TCG CAA TCG–3′ and 5′–GGG GAC CAC TTT GTA CAA GAA AGC TGG GTT TAC GCG ACC GGC GGC AGC ATA AAC GG–3′ (Cosmogenetech, Seoul, Korea). The synthesized gene and PCR products were reacted with the pDONR207 according to the manufacturer’s instructions. The created entry clone pENTR-hSCF164 was recombined with a destination vector (pDEST-His6, pDEST-PDIb’a’, and pDEST-HMGWA) to generate expression vectors. pENTR-His-hSCF248 was recombined with pDEST-His8 vector. Sequencing analysis confirmed the correct sequence for the expression vector (Macrogen, Daejeon, Korea).

### 4.3. Expression and Solubility of Fusion Proteins

Recombinant plasmids His-hSCF164, PDIb’a’-hSCF164, and MBP-hSCF164 were transformed into *E. coli* Origami 2(DE3). The His-hSCF248 plasmids were transformed into *E. coli* Origami 2(DE3) and BL21(DE3). To screen colonies expressing high levels of protein, transformed *E. coli* were grown on LB agar plates containing antibiotics (50 μg/mL ampicillin or 15 μg/mL gentamicin). The selected colonies were seeded in LB medium containing antibiotics and cultured at 37 °C with shaking at 180 rpm overnight. The overnight culture was then inoculated at 1:100 ratio into 4 mL of fresh LB medium, and the cells were incubated at 37 °C with shaking at 180 rpm. When OD_600_ reached 0.6–0.8, IPTG was added to a final concentration of 0.5 mM to induce recombinant protein expression. The cells were cultured at 30 °C for 4 h or at 18 °C for 18 h, with shaking at 180 rpm. The cultured cells were harvested by centrifugation at 3800× *g* for 30 min at 4 °C. The cell pellet was lysed on ice using an ultrasonic cell disruptor JY99-IIDN from Ningbo Scientz Biotechnology (Guangdong, China). Sonication was repeated for 40 cycles of 2 s followed by 2 s rest with sonication buffer (20 mM Tris-HCl, 1 mM EDTA, 20% glycerol (*v/v*), pH 8.0). The lysed cells were centrifuged at 17,000× *g* for 15 min at 4 °C to separate soluble and insoluble portions. The protein expression and solubility level were analyzed by 10% tricine SDS-PAGE gel and calculated using Gel Analyzer 19.1.

### 4.4. Purification and Tag Removal of Recombinant hSCF164 from E. coli

A single colony that was confirmed to express the MBP-hSCF was selected for the seed culture. This was inoculated in 500 mL of LB with 50 μg/mL ampicillin and grown overnight at 37 °C with shaking at 180 rpm. The overnight culture was then inoculated at 1:100 into fresh LB medium, and the cells were incubated at 37 °C with shaking at 180 rpm until the OD_600_ was approximately 0.6–0.8. IPTG was added to a final concentration of 0.5 mM, and incubation continued at 18 °C overnight. The induced cells were harvested by centrifugation at 3800× *g* for 30 min at 4 °C. After centrifugation, the supernatant was removed, and the cell pellets were stored at −20 °C.

The cell pellet was resuspended in 100 mL of buffer A (20 mM Tris-HCl, 500 mM NaCl, and 5% glycerol (*v/v*), pH 8.0) and disrupted by an ultrasonic cell disruptor. Lysed cells were then centrifuged at 23,000× *g* for 30 min at 4 °C. The supernatant was filtered through a 0.45 μm pore membrane. The filtered supernatant was applied to a 5 mL MBP Trap HP affinity column that was previously equilibrated with buffer A. After loading the supernatant, the column was washed with at least five CVs of buffer A to remove nonspecifically bound proteins. Buffer B (20 mM Tris-HCl, 500 mM NaCl, 20 mM maltose, and 5% glycerol (*v/v*), pH 8.0) was run through the column over five CVs to elute MBP–hSCF. The eluted sample was dialyzed in buffer A and was treated with homemade TEV protease (Addgene: 8827) to remove the MBP tag. The ratio of the fusion protein to TEV protease was 5:1 (*w/w*), and cleavage reaction was performed at 18 °C for 18 h.

The cleaved sample was filtered through a 0.45 μm pore membrane. To isolate the target protein, the sample was loaded in the 5 mL HisTrap FF column equilibrated with buffer A. The collected flowthrough including hSCF164 was dialyzed against buffer C (20 mM Tris-HCl, 5% glycerol (*v/v*), pH 8.0) using a 3.5 kDa MWCO membrane (Darien, IL, USA).

The dialyzed sample was applied to HiTrap Q HP column equilibrated with buffer C and then eluted with 10 CVs of elution buffer using an NaCl gradient (0–300 mM). Purified hSCF164 was concentrated using the 3 kDa Amicon Ultra from Merck Millipore by centrifuging at 3800× *g*; concentrated protein was dialyzed against PBS at pH 7.4 with 5% glycerol (*v/v*) using a 3.5 kDa MWCO membrane. All of the purification steps were analyzed by 10% tricine SDS-PAGE gel. Protein concentrations were measured by Bradford assay using bovine serum albumin as a standard.

### 4.5. Purification of Recombinant His-hSCF248 from E. coli

His-hSCF248 was expressed as described for hSCF164. The cell pellets were resuspended in 100 mL of binding buffer (20 mM Tris-HCl and 5% glycerol (*v/v*), pH 10) and lysed by an ultrasonic cell disruptor (2 s with a 28 s interval). Lysed cells were then centrifuged at 23,000× *g* for 30 min at 4 °C. After filtering the lysate supernatant through a 0.45 μm pore membrane, the fusion protein His-hSCF248 was loaded onto the 5 mL HiTrap Q HP column and eluted with 10 CVs of elution buffer using an NaCl gradient (0–1 M). The fraction was concentrated with 10 kDa Amicon Ultra from Merck Millipore by centrifuging at 3800× *g*. The concentrated sample was purified with GPC column, Hiload 16/600 Superdex 75 pg. The final product was dialyzed against PBS at pH 7.4 with 5% glycerol (*v/v*) using a 12 kDa MWCO membrane. All of the purification steps were analyzed by 10% tricine SDS-PAGE gel. Protein concentrations were measured by Bradford assay using bovine serum albumin as a standard.

### 4.6. Analysis in Electrophoresis and Silver Staining

Expression of the fusion protein and all protein samples in the purification steps was assessed via 10% tricine SDS-PAGE gel. The protein fractions were boiled for 10 min at 100 °C with 5× sample buffer (250 mM Tris-HCl, pH 6.8, 30% glycerol, 10% SDS, 0.01% bromophenol blue, and 300 mM DTT) to denature the proteins before loading on 10% tricine SDS-PAGE gels. Nonreducing conditions used DTT-free sample buffer. Separated proteins were stained with Coomassie brilliant blue R-250 solution. The expression and solubility levels (Equations 1 and 2) of the fusion proteins, and the purity of the target proteins were calculated using the Gel Analyzer program 19.1.

(1) $$\mathrm{Expression}\;\mathrm{level}\;=\frac{\;S\prime }{I\prime }$$

(2) $$\mathrm{Solubility}\;=\frac{\;S\prime \prime }{\left(S\prime \prime \;+\;P\text{'}\right)}$$

where *S*′ is the amount of fusion protein, *I*′ is the total amount of cellular protein after IPTG induction, *S*′′ is the amount of the fusion protein in the supernatant section (*S*), and *P*′ is the amount of the fusion protein in the pellet section (*P*).

Silver staining was performed with the Silver Stain Plus kit. Electrophorized SDS-PAGE gels were fixed with Fixative Enhancer Solution (50% methanol, 10% acetic acid, 10% fixative enhancer, and 30% distilled water, *v/v*) for 20 min. The gel was then rinsed with distilled water for 10 min twice. To visualize the bands of proteins, the gel was stained and developed in staining solutions according to protocol instructions. The developed gel was placed in 5% acetic acid solution for 15 min to stop the reaction.

### 4.7. Endotoxin Assay

Purified hSCF164 and His-hSCF248 were treated with Triton X-114 to remove endotoxin. Samples treated with 1% Triton X-114 were mixed by inversion for 1 h at 4 °C. Thereafter, the samples were incubated for 15 min at room temperature. Once a sample became opaque, this was centrifuged at 23,000× *g* for 15 min at room temperature and then the supernatant was removed. The Toxin Sensor Chromogenic LAL Endotoxin Assay Kit was used to quantify the endpoint endotoxin level. The samples were prepared by diluting to 1 μg/mL with LAL reagent water. LAL reagents 100 (μL) were added to 100 μL samples and standards of endotoxin-free vials, which were then incubated at 37 °C for 30 min. Samples were then treated with 100 μL of chromogenic substrate to each vial and incubated at 37 °C for 6 min. After that, 500 μL of color-stabilizer #1, 500 μL of color-stabilizer #2, and 500 μL of color-stabilizer #3 were added to each vial in order. Each reaction had absorbance read at 545 nm by a spectrophotometer.

### 4.8. In Vitro Activity Assay

The biological activity of purified hSCF164 and His-hSCF248 was measured by the effect on proliferation of the human erythrocytic TF-1 cell line. TF-1 cells were grown in RPMI 1640 medium supplemented with 2 ng/mL GM-CSF, 10% FBS, and 1% penicillin and streptomycin at 37 °C in a CO_2_ incubator. TF-1 cells were washed with 1× PBS to remove all cytokines before the activity test. Cells (1 × 10^4^ per well) were added to a 96-well plate and treated with different concentrations of hSCF164 and His-hSCF248 for 12 h at 37 °C and 5% CO_2_. The test medium was similar to the growth medium but lacked the 2 ng/mL GM-CSF. The final concentration range of purified hSCF164 was from 0.01 to 1000 ng/mL, and that of purified His-hSCF248 was from 0.01 to 1000 pg/mL. All of the concentration conditions were repeated in triplicate. After 12 h incubation, 10 μL of CCK-8 reagent was added to each well, and the plate was incubated for 3 h at 37 °C. The absorbance of the sample was measured at 450 nm with a microplate reader. The data were processed using following Equation (3) and Microsoft Excel software:

(3) $$\;Re=Bl+\frac{Max-Bl}{1+{\left(\frac{EC50}{conc.}\right)}^{Hs}}$$

where *Re* is response of the cells, *Bl* is the baseline at low concentration, *Max* is the maximum response, *conc.* is the concentration of the protein, and *Hs* is the Hill coefficient of stimulation.

### 4.9. RNA Preparation and Real-Time Quantitative PCR

TF-1 cells were cultured in RPMI 1640 medium supplemented with 10% FBS and 1% penicillin and streptomycin at different concentration (from 0.01 to 1000 ng/mL for hSCF164 and from 0.01 to 1,000 pg/mL for His-hSCF248). TF-1 cells were harvested by centrifugation at 200× *g* for 5 min at 4 °C and then treated with Trizol to extract RNA. The isolated RNA samples were reverse-transcribed using the Reverse Transcription Master Premix according to the manufacturer’s instructions. To analyze the expression of *c-Myc*, the following primers were used: *c-Myc* forward 5′–AAT GAA AAG GCC CCC AAG GT–3′, reverse 5′–GTC GTT TCC GCA ACA AGT CC–3′; RPL forward 5′–GTG GTT CCT GCA TGA AGA CAG TG–3′, reverse 5′–TTC TGA TGG CGG ACT TTA CCG–3′ For quantitative PCR analysis, a homemade reaction mixture was made using homemade buffer (500 mM Tris-HCl, pH 8.5, 160 mM (NH_4_)_2_SO_4_, 25 mM MgCl_2_, 1% Tween-20, and 1 mg/mL BSA) and 0.06 mM dNTP, 0.4× SYBR Green I, 16.8 ng/μL Taq DNA polymerase, 0.5 μM ROX dye (AATbioquest, CA, USA), 5 pmol of each primer, and 100 ng/μL cDNA. Real-time PCR system amplification conditions were as follows: hold at 95 °C for 5 min, followed by 35 cycles of 95 °C for 10 s, 65 °C for 10 s, and 72 °C for 30 s. *RPL* was used as an internal control gene to normalize for RNA quantity. The relative gene-specific expression was calculated using the 2^−ΔΔCt^ method after normalization to *RPL* expression.

### 4.10. Cell Cycle Assay

TF-1 cells were treated with different concentrations of hSCF for 12 h at 37 °C and 5% CO_2_. After 12 h incubation, the cells were harvested by centrifugation at 200× *g* for 5 min at 4 °C and fixed in 70% ethanol for 15 h at 4 °C. Cells were then incubated in 500 µL of propidium iodide (PI) staining solution (50 µg/mL PI, 0.1 mg/mL RNase A, and 0.05% Triton X-100 in PBS) for 40 min at 37 °C. The samples were read using FACS canto II and BD FACS Diva Software (v8.02).

### 4.11. Protein Stability Assay

The soluble fraction of *E. coli* expressing His-hSCF248 was divided into nine samples. The pH of eight samples were adjusted from pH 3 to pH 10 by pH 1 increment. After 1 h incubation at RT, the samples were centrifuged at 17,000× *g* for 15 min at 4 °C. The amounts of remaining proteins in the soluble fraction were measured with the Bradford method.

### 4.12. Statistics

The statistical significance of the responses was performed by one-way ANOVA and Dunnett post hoc in GraphPad Prism (v5.0). Values of *p* < 0.05 were considered to indicate statistical significance. All data are presented as the mean ± SEM of *n* ≥ 3 independent experiments.

## 5. Conclusions

This study demonstrates that recombinant His-hSCF248 is expressed in a soluble form in *E. coli* cytoplasm and has greater activity than the recombinant hSCF164. Additionally, the soluble expression of hSCF164 is enhanced by fusion with MBP.

## Figures and Tables

**Table 1:** Expression levels and solubilities of His-hSCF164, PDIb’a’-hSCF164, and MBP-hSCF164 in Origami 2(DE3) from triplicated experiments.

		Expression Level (%)		Solubility (%)	
	**Tag**	**18 °C**	**30 °C**	**18 °C**	**30 °C**
	His6	16 ± 0.23	38.2 ± 0.5	59.4 ± 8	12.1 ± 5.2
hSCF164	PDIb’a’	34.2 ± 1.1	22.1 ± 3.8	95.6 ± 2.9	4.9 ± 1.6
	MBP	35 ± 3	48.2 ± 5.2	96.8 ± 2.3	84.7 ± 11.2

**Table 2:** Expression levels and solubilities of His-hSCF248 in Origami 2(DE3) and BL21(DE3) from triplicated experiments.

		Expression Level (%)		Solubility (%)	
	**Strain**	**18 °C**	**30 °C**	**18 °C**	**30 °C**
His-hSCF248	Origami 2(DE3)	29 ± 10.9	36.7 ± 5.9	35.1 ± 14.4	6.2 ± 3.2
	BL21(DE3)	56.3 ± 5.2	62.5 ± 4.1	61.2 ± 17.9	9.7 ± 3.5

**Table 3:** Purification yields of hSCF164.

**Purification Step**	**Total Protein (mg)**	**Purity (%)**	**hSCF164 (mg)**	**Yield (%)**
Supernatant	61.44	35.5	21.8	100
Dextrin	9.90	86.3	8.54	39.2
IMAC	3.66	54.6	1.99	23.3
Anion exchange	0.24	99.6	0.24	12.0

The results were derived from 1.2 g of wet cell weight from 500 mL of cultured cells.

**Table 4:** The purification yields of His-hSCF248.

**Purification Step**	**Total Protein (mg)**	**Purity (%)**	**His-hSCF248 (mg)**	**Yield (%)**
Supernatant	60.8	27.4	16.65	100
Anion exchange	21.9	48.5	10.02	60.2
Gel filtration	2.5	96.3	2.40	23.9

The results were derived from 1.6 g of wet cell weight in 500 mL of cultured cells.

## Data Availability

The datasets generated and analyzed during the present study are available from the corresponding author on reasonable request.

## Supplementary Materials

The following are available online at https://www.mdpi.com/article/10.3390/ijms22126361/s1.
