# Supplementary figures and images for: Novel Bacterial Production of Two Different Bioactive Forms of Human Stem-Cell Factor
Source: Int J Mol Sci. 2021 Jun 14;22(12):6361. doi: 10.3390/ijms22126361 (PMC8232154; doi:10.3390/ijms22126361)

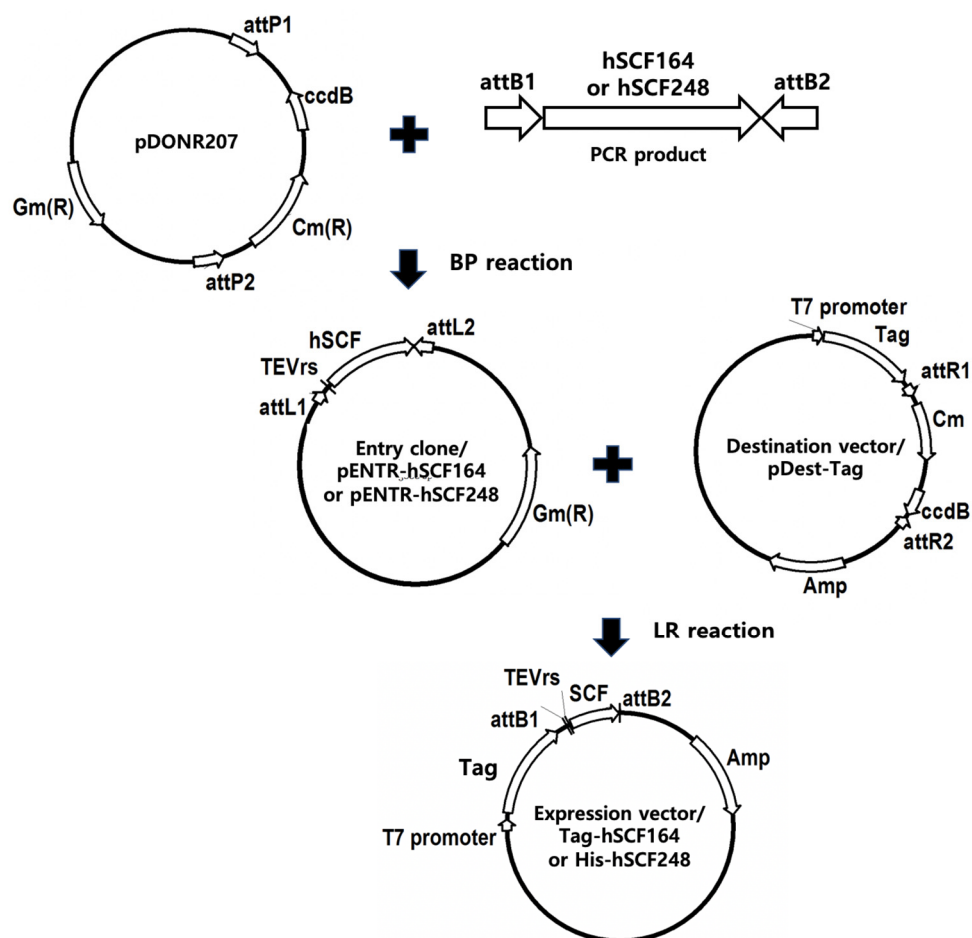

**Supplementary Figure S1.** Summary of the expression vector cloning process.

Supplement: Supplementary file 1 [file ijms-22-06361-s001.zip › ijms-1219990-supplementary.pdf]
